# Amyloid Accumulation in the Toxic Nodule of the Thyroid Gland in a Patient with End Stage Renal Failure

**DOI:** 10.1155/2012/741754

**Published:** 2012-10-23

**Authors:** Umut Mousa, Sema Aktas, Halit Uner

**Affiliations:** ^1^Department of Endocrinology and Metabolism, Baskent University Faculty of Medicine, 06500 Ankara, Turkey; ^2^Department of General Surgery, Baskent University Faculty of Medicine, 06500 Ankara, Turkey; ^3^Department of Pathology, Baskent University Faculty of Medicine, 06500 Ankara, Turkey

## Abstract

Amyloidosis is characterized by accumulation of amorphous, proteinaceous material in various organs and tissues of the body. Amyloid may accumulate in the thyroid gland in cases of medullary thyroid carcinoma and systemic amyloidosis. Amyloid accumulates extracellularly in the thyroid parenchyma and disrupts the normal follicular patterns. Most of the cases reported up to now were clinically euthyroid, but many presentation forms and overlaps have been reported. Herein we present a patient with toxic nodular goiter with amyloid deposition in the toxic nodule as well as the remaining thyroid tissue.

## 1. Introduction

Amyloidosis is characterized by accumulation of amorphous, proteinaceous material in various organs and tissues of the body [1]. The mechanism is not clearly defined.

Amyloidosis can be primary or secondary according to the etiology. Secondary amyloidosis occurs in chronic inflammatory states such as rheumatoid arthritis, Chrohn's disease, osteomyelitis and tuberculosis. Consequently, almost any disease associated with chronic inflammation of whatever etiology is liable to amyloidosis complications [2]. Serum amyloid protein (SAA) is responsible for secondary amyloidosis [3, 4]. Amyloid deposit in the thyroid gland was first reported by von Rokitansky in 1855 [5]. Amyloid may accumulate in the thyroid gland in cases of medullary thyroid carcinoma and systemic amyloidosis. Diffuse, clinically apparent enlargement of the thyroid gland due to widespread amyloid deposit is a rare occurrence. In 1904 von Eisenberg introduced the term “amyloid goiter” into the literature. It is defined by the presence of amyloid within the thyroid gland in such quantities as to produce clinically apparent enlargement of the gland [6]. Most of the cases reported up to now were clinically euthyroid. Patients with hyperthyroxinemia and hypothyroidism have been reported. Some patients resembling subacute thyroiditis have also been reported. Systemic amyloidosis may occur in patients with kidney failure. In patients who have been on a hemodialysis or peritoneal dialysis program for more than five years, *β*2 microglobulin builds up forming deposits. This is named as dialysis-related amyloidosis and often occurs around joints. This substance is normally cleared by the kidneys but cannot be removed by dialysis membranes [2]. Herein we present a patient with toxic nodular goiter with amyloid deposition in the toxic nodule as well as the remaining thyroid tissue.

## 2. Case Report

A 52-year-old female patient with known chronic renal failure and on a routine hemodialysis program for seven years was hospitalized for prerenal transplant evaluation.

**Figure 1:**
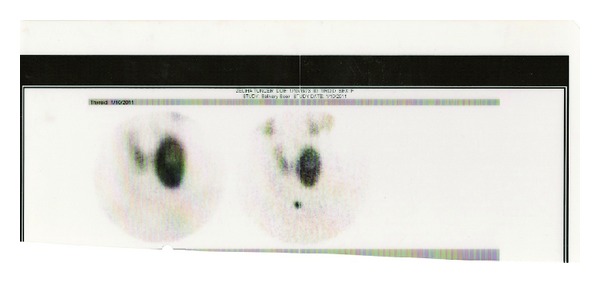
Thyroid scan reveals the active nodule of the left lobe and suppression in the remaining thyroid tissue.

**Figure 2:**
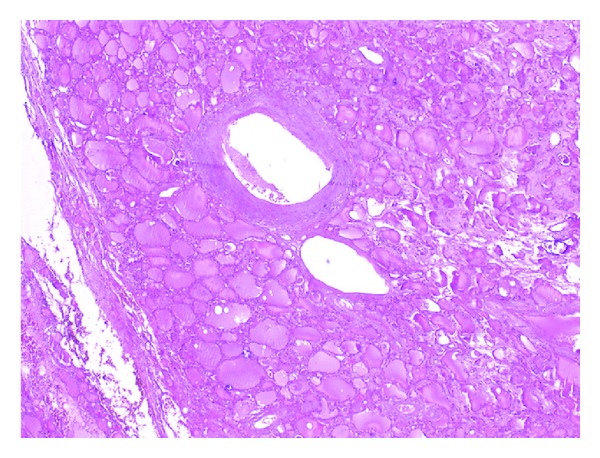
The thyroid nodule is seen apparently seperated from the surrounding thyroid tissue. The nodule shows follicular patterns of different sizes not compressing the surrounding tissue. Eosinophilic and hyaleneceous amorphous substance accumulation is seen in the arterial wall and interstitium compatible with amyloid (H & E).

**Figure 3:**
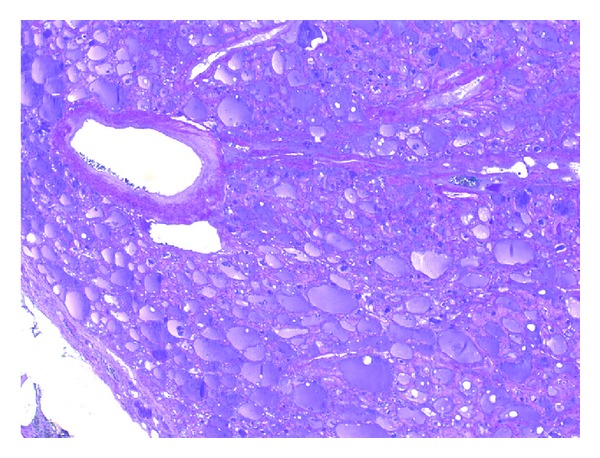
Pink-colored substance accumulation compatible with amyloidosis is seen in the arterial wall inside the nodule together with the parafollicular area and interstitium (crystal violet).

**Figure 4:**
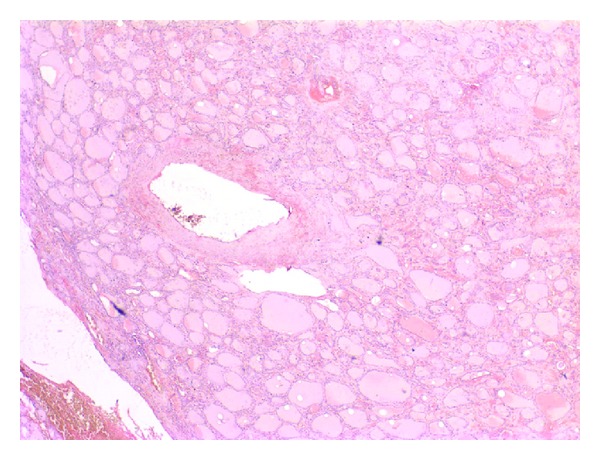
Red-colored amorphous substance accumulation compatible with amyloid is seen in the interstitium within the nodule together with the arterial wall compressing parafollicular cells and thyroid epithelial cells (congo red).

**Figure 5:**
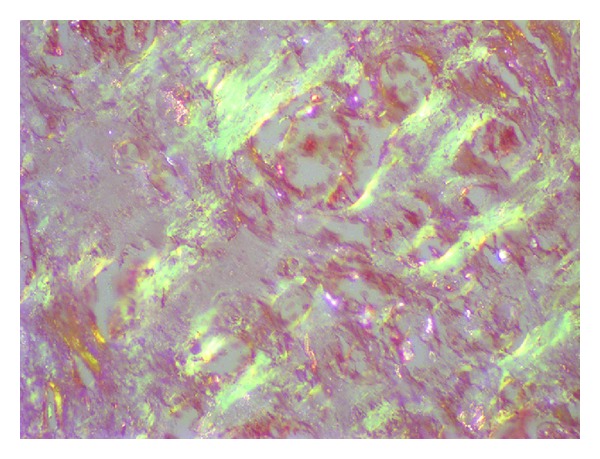
Material was confirmed apple green under polarized microscope.

According to her medical history, she was completely healthy seven years ago and her kidneys deteriorated after usage of an antibiotic drug. Routine thyroid function tests were compatible with subclinical hyperthyroidism (T_4_ 17 pmol/L (9–20), T_3_ 6.2 pmol/L (3.5–8), TSH 0.20 *μ*IU/mL (0.35–5.1)) so a thyroid ultrasonography was performed revealing a 55 × 36 × 25 mm hypoechoic nodule almost completely filling the left lobe. Anti-thyroglobulin and anti-thyroid peroxidase autoantibodies were negative. She had no compressive symptoms. Increased activity in the nodule and suppression in the remaining thyroid tissue were reported in her thyroid scan by technetium (Figure 1). Fine needle aspiration cytology (FNAC) revealed normal thyrocytes and was reported as benign. She was diagnosed as toxic uninodular goiter, and total thyroidectomy was performed. The macroscopic specimen revealed amyloidosis in the right lobe, amyloidosis in the nodule, interstitial area, and perivascular areas in the left lobe of the thyroid gland (Figures 2, 3, 4, and 5). She is now on 100 mcg levothyroxine replacement therapy and is being prepared for renal transplantation.

## 3. Discussion

It is generally accepted that some degree of deposition of amyloid in the thyroid gland can be detected in more than 80% of patients suffering from secondary amyloidosis and approximately 50% of those affected by primary amyloidosis [7]. Usually it is diagnosed in autopsies and macroscopic specimens, but recent studies have focused on FNAC in diagnosis. Amyloid goiter is a rarer entity. Amyloid accumulates extracellularly in the thyroid parenchyme and disrupts the normal follicular patterns [8, 9].

Most patients are clinically euthyroid, but many different presentation forms have been reported. The thyroid gland can be soft, hard, diffuse or nodular in character according to the amount of amyloid deposited [3]. Kimura et al. reported 10 thyroidal involvements out of 30 cases with systemic amyloidosis. Nine of these patients had some kind of thyroid dysfunction. 5 patients had hypothyroidism, 2 patients had low T3 syndrome, 1 patient had subacute thyroiditis-like syndrome, and 1 had coexisting Graves disease. Five out of the nine patients had positive thyroid autoantibodies [1].

The etiology of amyloidosis in our patient was uncertain. Several presentations and case reports have been reported about dialysis-related amyloidosis. One case was even reported as carpal tunnel syndrome being the presenting feature [10]. Thyroid involvement is not common in dialysis-related amyloidosis. Musculoskeletal manifestations are more prominent in this type of amyloidosis which were absent in our patient. She had been on a peritoneal dialysis program for five years prior to switching to hemodialysis. She reported being hospitalized for peritonitis two times. Repeated peritonitis attacks may have contributed to her developing secondary sistemic amyloidosis. Maybe systemic amyloidosis was the etiology for chronic renal failure in the first place, but kidney biopsy had never been performed.

Our patient had also toxic nodular goiter. The interesting fact in our case was that the nodule was not an amyloid nodule but a toxic nodule with intranodular amyloid deposition. Amyloid was also deposited in the thyroid parenchyme. The nodule was confirmed active with scintigraphy showing that the amyloid deposit did not interfere with technetium. Since amyloid leads to dysfunctioning of the deposited organ, the expected outcome was thyroidal enlargement and primary hypothyroidism. A previous letter by Tokyol et al. revealed similar findings. They argued that hyperthyroidism could be a secondary response of the thyroid gland to interstitial infiltration by amyloid material [11].

It should be noted that amyloid can deposit in all organs and tissues of the body including the thyroid gland. Amyloid accumulation in the thyroid gland does not usually cause thyroid dysfunction and most patients are euthyroid. The importance of this case is that it proves that amyloid can accumulate in thyroid nodules and even in toxic nodules. Whether amyloid deposition contributes to developing thyroidal diseases other than goiter and hypothyroidism needs further research and experience.
